# A Survey of Physicians' Perception of the Use and Effectiveness of Diagnostic and Therapeutic Procedures in Chronic Cough Patients

**DOI:** 10.1007/s00408-021-00475-1

**Published:** 2021-09-17

**Authors:** Luis Puente-Maestu, Jesús Molina-París, Juan A. Trigueros, J. Tomás Gómez-Sáenz, Luis Cea-Calvo, Sabela Fernández, Marta Sánchez-Jareño, Javier Domínguez-Ortega

**Affiliations:** 1 Pulmonology Department, University Hospital Gregorio Marañón, Madrid, Spain; 2 Francia Healthcare Center, Fuenlabrada, Madrid, Spain; 3 Menasalbas Healthcare Center, Toledo, Spain; 4 Nájera Healthcare Center, La Rioja, Spain; 5 Medical Affairs Department, MSD, Madrid, Spain; 6 Department of Allergy, University Hospital La Paz, Institute for Health Research IdiPAZ, Madrid2 CIBER of Respiratory Diseases, CIBERES, Madrid, Spain; 7 Servicio de Neumología. Hospital Universitario Gregorio Marañón, c/ Dr. Esquerdo 46, 28007 Madrid, Spain

**Keywords:** Chronic cough, Diagnosis, Primary care, Pulmonology, Allergology, Survey

## Abstract

**Purpose:**

The aim of this study was to understand the perception of family physicians, pulmonologists, and allergists with respect to diagnostic tests performed on patients with chronic cough and treatments prescribed to patients with refractory or unexplained chronic cough. We also assessed how these health professionals perceived the effectiveness of these treatments.

**Methods:**

An anonymous survey was distributed by the scientific societies SEPAR, SEAIC, SEMERGEN, semFYC, and SEMG. Respondents were asked how often they perform diagnostic tests and prescribe treatments (responses from 1 = never to 10 = always) and how they perceived the effectiveness of the drugs used (from 1 = not at all to 10 = very effective). The correlation between perceived effectiveness and frequency of prescription was analyzed.

**Results:**

The respondents comprised 620 family physicians, 92 pulmonologists, and 62 allergists. The most frequently performed diagnostic tests were chest x-ray and, among pulmonologists and allergists, simple spirometry and bronchodilator tests. The most frequently prescribed drugs were bronchodilators (percentages scoring 8–10 for each specialty: 43.2%, 42.4%, and 56.5%; *p* = 0.127), inhaled corticosteroids (36.9%, 55.4%, and 54.8%; *p* < 0.001), and antitussives (family physicians, 33.4%). Regarding perceived effectiveness, only bronchodilators, inhaled or oral corticosteroids, and opioids obtained a median effectiveness score > 5 (between 6 and 7). Correlation coefficients (ρ^2^) suggested that approximately 45% of prescription was related to perceived effectiveness.

**Conclusion:**

Although chronic cough is a common problem, diagnosis and treatment differ among specialists. The perceived effectiveness of drugs is generally low.

**Supplementary Information:**

The online version contains supplementary material available at 10.1007/s00408-021-00475-1.

## Introduction

Cough is a protective reflex that prevents aspiration and helps to clear the airways. However, when cough lasts over time, it can prove disabling [1]. Chronic cough affects approximately 10% of adults [2], but studies apply different durations. The most recent guidelines define chronic cough as cough lasting more than 8 weeks [3].

In its 2020 guidelines on the management of chronic cough, the European Respiratory Society (ERS) describes the most characteristic phenotypes of chronic cough (asthmatic cough/eosinophilic bronchitis, reflux cough, postnasal drip syndrome/upper airway cough syndrome, and iatrogenic cough) [3] in order to establish “treatable traits”. However, even if a diagnosis is reached and appropriate treatment administered, cough persists in many patients (refractory chronic cough), whereas in others, no specific cause is identified after an exhaustive diagnostic work-up (unexplained chronic cough). A feature common to many patients with chronic cough is hypersensitivity to the cough reflex, which induces cough on exposure to low levels of tussive stimuli (hypertussia) or to stimuli that do not usually produce cough (allotussia). In addition, the response to treatments targeting the underlying disease is often inadequate [4]. Recent studies on the pathophysiological mechanisms involved in the development of chronic cough have demonstrated the participation of underlying neurophysiologic abnormalities, thus leading this condition to be considered a specific entity and not a mere symptom [3, 5–7].

The assessment of chronic cough relies on an evaluation of the characteristics of cough phenotypes. The ERS guidelines recommend spirometry and chest x-ray in the initial evaluation [3]. In order to identify treatable traits of chronic cough, more evaluations should be performed to target asthma, eosinophilic bronchitis, gastroesophageal reflux, altered esophageal motility, and rhinosinusitis. However, even after an exhaustive clinical evaluation, it is sometimes impossible to identify a probable treatable trait or a specific underlying disease.

In this context, several therapeutic options have been used, sometimes empirically, to treat refractory or unexplained cough even when evidence is scarce for some drugs [3], resulting in heterogeneity in the diagnosis and treatment of chronic cough, not only between different specialists, but also within the same specialty. In order to obtain a picture of diagnosis and management of chronic cough (and, more specifically, refractory or unexplained chronic cough) in Spain by family physicians, pulmonologists, and allergists, we performed a survey through their respective scientific societies.

In this study, we describe the diagnostic work-up preferred by these specialists in patients with chronic cough, the drugs prescribed by physicians to patients with refractory or unexplained chronic cough, their perception of the effectiveness of these treatments, and the correlation between perceived effectiveness and prescription of different drug families.

## Methods

### Study Design

The study was based on a cross-sectional survey sent to family physicians, pulmonologists, and allergists. The survey was designed jointly with members of the scientific societies of each specialty who had experience in the treatment of chronic cough (list provided in the acknowledgements section), and addressed to physicians working mainly in the Spanish public national health system. This public system covers all the Spanish population, and patients’ first access to the system is done though the primary care physician, which is the first physician to assess chronic cough patients most of the time. Additionally, a small percentage of population is covered by private health care insurances, which allow a more direct access to specialists.

The survey included a section on the diagnosis of patients with chronic cough and a section about treatment of patients with refractory or unexplained chronic cough, with questions on the drug families prescribed to affected patients and physicians’ perception of their effectiveness. Physicians were offered a list of diagnostic procedures for chronic cough and therapeutic agents for refractory or unexplained cough and were prompted to assign a frequency score according to their clinical practice on a Likert scale from 1 (never performed/prescribed) to 10 (always performed/prescribed). The perceived effectiveness of each therapeutic class was evaluated by asking physicians to assign a value running from 1 (not effective at all) to 10 (very effective). As physicians were not asked to order, they could assign the same score to different diagnostic tests or therapeutic agents. The complete survey is provided as Supplementary material.

The content of the survey was hosted on a web platform, which physicians could access by invitation from their scientific societies through a link sent by email. The number of physicians to which the survey was sent was estimated to be about 32,000 family physicians, 2,250 pulmonologists, and 1,500 allergists. The exact number of each group cannot be calculated, as one physician can be member of more than one scientific society.

Physicians’ consent was given on agreeing to participate and on completing the survey voluntarily and anonymously. Physicians responded based on their experience and perceptions of their clinical practice, without review of clinical data or patient registries.

### Statistical Analysis

Responders and their responses were evaluated using measures of central tendency and dispersion for the numerical variables (mean and standard deviation, median and interquartile range) and percentages for the categorical variables.

The results for frequency of indication of diagnostic tests, prescription of drug families, and perceived effectiveness were expressed using the median (interquartile range) and the percentage of physicians who assigned the highest scores (8–10) to each test or drug family. The differences in percentage between the 3 specialties were evaluated using the chi-square test.

The correlation between perceived effectiveness of the different drug families and the frequency of prescription was evaluated by calculating the Spearman’s rho (ρ) correlation coefficients and their squares (ρ^2^) for each drug family and specialty separately. In addition, a global correlation study was performed for all the drugs grouped by summing the individual scores of each drug for each specialty and for all the specialists grouped together.

## Results

The survey ran for 6 weeks, from early February to mid-March 2020 and was completed by 620 family physicians, 92 pulmonologists, and 62 allergists. Of these, 470 were women (60.7%) and 304 were men (39.3%). Participation rate was approximately 1.9% of family physicians, 4.1% of pulmonologists, and 4.1% of allergists. Most of the respondents (89.9%) worked only in the public national health system, 5.3% in both public and private clinics, and only 5.3% in private sector only.

The median number of patients with chronic cough (with or without underlying disease) the participants reported having seen at their clinics during the week before completing the survey was 5 for family physicians, 7 for pulmonologists, and 4 for allergists. The percentages of respondents reporting that they frequently/very frequently see patients with chronic cough without underlying disease were 41.8% of primary care physicians, 43.5% of pulmonologists, and 58.1% of allergists.

The only guideline for the management of chronic cough patients that was significantly followed was the Spanish guideline from the Spanish Society of Respiratory Medicine (SEPAR), followed by 87.0% of pulmonologists, 40.3% allergists, and 49.0% of primary care physicians. Other guidelines (they were asked specifically for European, British, and American guidelines) were seldom used (13.8%, 5.8%, and 6.7%, respectively, frequencies higher among pulmonologists).

Table 1 shows the frequency (scored from 1 [never] to 10 [always]) with which the 3 groups of specialists reported performing specific diagnostic tests to study patients with chronic cough. They all scored chest x-ray, simple spirometry, and the bronchodilator test as most frequently used. Plain x-ray was the most common test among family physicians and pulmonologists, whereas simple spirometry was the most common among allergists. Pulmonologists and allergists reported more frequently using more specific tests, such as fraction of exhaled nitric oxide (FeNO) or determination of total and specific IgE (allergists) (Table 1).

**Table 1:** Diagnostic tests performed for the study of patients with chronic cough: Scores for frequency of indication

	Median (IQR)			Percentage with the highest score (8–10), n (%)			
	Family physicians# (*n* = 620)	Pulmonologists (*n* = 92)	Allergists (*n* = 62)	Family physicians# (*n* = 620)	Pulmonologists (*n* = 92)	Allergists (*n* = 62)	*p*-value*
Chest x-ray	9 (7–10)	10 (10–10)	8 (5–10)	432 (69.7)	88 (95.7)	31 (50.0)	< 0.001
Simple spirometry	6 (3–8)	10 (9–10)	10 (10–10)	234 (37.7)	75 (81.5)	55 (88.7)	< 0.001
Bronchodilator test	8 (5–9)	10 (10–10)	10 (9–10)	311 (50.2)	87 (94.6)	52 (83.9)	< 0.001
Methacholine test	1 (1–1)	5 (3–7)	5 (2–7)	7 (1.1)	13 (14.1)	15 (24.2)	< 0.001
FeNO test	1 (1–1)	7 (3–10)	8 (3–10)	7 (1.1)	43 (46.7)	35 (56.5)	< 0.001
Capsaicin test	1 (1–1)	1 (1–1)	1 (1–1)	5 (0.8)	1 (1.1)	1 (1.6)	0.799
Complete blood count	8 (5–9)	8 (5–10)	8 (5–10)	310 (50.0)	48 (52.2)	31 (50.0)	0.926
Total IgE	6 (3–8)	5 (3–7)	9 (6–10)	198 (31.9)	45 (48.9)	36 (58.1)	< 0.001
Specific IgE	4 (1–7)	5 (4–7)	8 (5–10)	113 (18.2)	21 (22.8)	31 (50.0)	< 0.001
*Chlamydia/Mycoplasma* serology	1 (1–3)	2 (1–3)	2 (1–5)	24 (3.9)	2 (2.2)	5 (8.1)	0.175
Esophageal pH monitoring	1 (1–1)	3 (2–5)	3 (1–5)	17 (2.7)	5 (5.4)	6 (9.7)	0.012

Physicians scored the frequency with which they indicate each test on a scale ranging from 1 = “Never” to 10 = “Always”; IQR, interquartile range; FeNO, fractional exhaled nitric oxide

^*^p-values refer to the differences in the percentages with the highest score (8–10) across specialties

^#^Although there might be variations from center to center, in general primary care physicians have no access or limited access to the following diagnostic tests: methacholine test, FeNO test, capsaicin test, specific Ig E, and esophageal pH monitoring. These tests are performed by pulmonologists and allergists (methacholine test, FeNO test, capsaicin test) or by gastroenterologists (esophageal pH monitoring)

The most commonly prescribed drugs for patients with refractory or unexplained chronic cough by the 3 specialties were inhaled bronchodilators and inhaled corticosteroids (Table 2). The drugs with the highest prescription rates (score 8–10) for allergists were inhaled bronchodilators (56.5%) and inhaled corticosteroids (54.8%), whereas for pulmonologists these were inhaled corticosteroids (55.4%) followed by inhaled bronchodilators (42.4%). In the case of family physicians, the drugs with the highest prescription rates (score 8–10) were inhaled bronchodilators (43.2%), inhaled corticosteroids (36.9%), and antitussives (33.4%). As for opioid derivatives, the group with the highest prescription rate (8–10) was family physicians (22.6%), followed by pulmonologists (17.4%) and allergists (6.5%) (Table 2).

**Table 2:** Scores assigned to the frequency of prescription of different treatments for refractory or unexplained chronic cough

	Median (IQR)			Percentage scoring highest (8–10), n (%)			
	Family physicians (*n* = 620)	Pulmonologists (*n* = 92)	Allergists (*n* = 62)	Family physicians (*n* = 620)	Pulmonologists (*n* = 92)	Allergists (*n* = 62)	*p*-value*
Antitussives^(1)^	6 (3–8)	4 (2–7)	2 (1–4)	207 (33.4)	17 (18.5)	2 (3.2)	< 0.001
Opioids^(2)^	5 (3–7)	4 (2–7)	2 (1–4)	140 (22.6)	16 (17.4)	4 (6.5)	0.008
Mucolytics^(3)^	5 (2–7)	3 (1–5)	2 (1–4)	127 (20.5)	8 (8.7)	2 (3.2)	< 0.001
Levodropropizine	1 (1–3)	1 (1–2)	1 (1–1)	19 (3.1)	3 (3.3)	0 (0.0)	0.371
Terpene derivatives	1 (1–1)	1 (1–1)	1 (1–1)	6 (1.0)	1 (1.0)	0 (0.0)	0.731
Antihistamines	6 (4–8)	5 (4–8)	6 (5–8)	186 (30.0)	23 (25.0)	18 (29.0)	0.616
Inhaled corticosteroids	7 (5–8)	8 (6–9)	8 (6–9)	229 (36.9)	51 (55.4)	34 (54.8)	< 0.001
Oral corticosteroids	4 (2–6)	3 (2–6)	3 (1–5)	85 (13.7)	10 (10.9)	4 (6.5)	0.222
Inhaled bronchodilators	7 (5–8)	7 (5–8)	8 (5–9)	268 (43.2)	39 (42.4)	35 (56.5)	0.127
Neuromodulators^(4)^	1 (1–2)	2 (1–4)	1 (1–2)	5 (0.8)	6 (6.5)	2 (3.2)	< 0.001

Physicians scored the frequency of prescription of each drug on a scale ranging from 1 = “Never” to 10 = “Always”; IQR, interquartile range. (1) dextromethorphan, cloperastine; (2) codeine, dimemorfan; (3) guaifenesin, acetylcysteine, ambroxol; (4) gabapentin, pregabalin

^*^p-values refer to the differences in the percentages with the highest score (8–10) across specialties

The perceived effectiveness of the drugs (from 1 [not at all] to 10 [very effective]) for chronic refractory or unexplained cough is shown in Table 3. Among the 3 specialties, the therapeutic families with a mean effectiveness score greater than 5 (median, 6–7) and percentage of physicians who assigned the highest efficacy scores (8–10) greater than 25% were inhaled bronchodilators, inhaled corticosteroids, and oral corticosteroids. Opioids also had a high score among family physicians and pulmonologists. The lowest scores (1–3) recorded were assigned to neuromodulators, mucolytic agents, levodropropizine, and terpene derivatives (Table 3).

**Table 3:** Scores assigned to the perceived effectiveness of the different treatments for refractory or unexplained chronic cough

	Median (IQR)			Percentage scoring highest (8–10), n (%)				Percentage scoring lowest (1–3), n (%)			
	Family physicians (*n* = 620)	Pulmonologists (*n* = 92)	Allergists (*n* = 62)	Family physicians (*n* = 620)	Pulmonologists (*n* = 92)	Allergists (*n* = 62)	p-value*	Family physicians (*n* = 620)	Pulmonologists (*n* = 92)	Allergists (*n* = 62)	*p*-value*
Antitussives^(1)^	5 (3–6)	4 (3–6)	4 (2–5)	74 (11.9)	10 (10.9)	1 (1.6)	0.046	228 (36.8)	36 (39.1)	30 (48.4)	0.193
Opioids^(2)^	6 (4–8)	6 (4–8)	4 (2–6)	158 (25.5)	24 (26.1)	5 (8.1)	0.008	123 (19.8)	16 (17.4)	27 (43.5)	< 0.001
Mucolytics^(3)^	3 (1–5)	3 (1–5)	2 (1–5)	48 (7.7)	3 (3.3)	1 (1.6)	0.068	340 (54.8)	61 (66.3)	41 (66.1)	0.038
Levodropropizine	2 (1–4)	1 (1–3)	1 (1–2)	12 (1.9)	1 (1.1)	0 (0.0)	0.472	452 (72.9)	73 (79.3)	52 (83.9)	0.089
Terpene derivatives	1 (1–1)	1 (1–2)	1 (1–1)	6 (1.0)	0 (0.0)	0 (0.0)	0.472	561 (90.5)	82 (89.1)	57 (91.9)	0.842
Antihistamines	5 (4–7)	5 (3–7)	5 (4–7)	106 (17.1)	12 (13.0)	7 (11.3)	0.342	122 (19.7)	30 (32.6)	12 (19.4)	0.017
Inhaled corticosteroids	7 (5–8)	7 (4–8)	7 (5–8)	215 (34.7)	33 (35.9)	19 (30.6)	0.782	65 (10.5)	19 (20.7)	5 (8.1)	0.012
Oral corticosteroids	6 (3–8)	6 (3–8)	7 (4–8)	171 (27.6)	28 (30.4)	21 (33.9)	0.521	170 (27.4)	27 (29.3)	11 (17.7)	0.222
Inhaled bronchodilators	7 (5–8)	6 (4–8)	7 (4–8)	241 (38.9)	23 (25.0)	17 (27.4)	0.011	60 (9.7)	20 (21.7)	11 (17.7)	0.001
Neuromodulators ^(4)^	1 (1–3)	3 (1–5)	1 (1–5)	12 (1.9)	5 (5.4)	4 (6.5)	0.026	493 (79.5)	54 (58.7)	45 (72.6)	< 0.001

Physicians scored the effectiveness of each drug on a scale ranging from 1 = “Not at all” to 10 = “Very effective”; IQR, interquartile range. (1) dextromethorphan, cloperastine; (2) codeine, dimemorfan; (3) guaifenesin, acetylcysteine, ambroxol; (4) gabapentin, pregabalin

^*^p-values refer to the differences in the percentages with the highest score (8–10) or with the lowest scores (1–3) across specialties across specialties

**Fig. 1:**
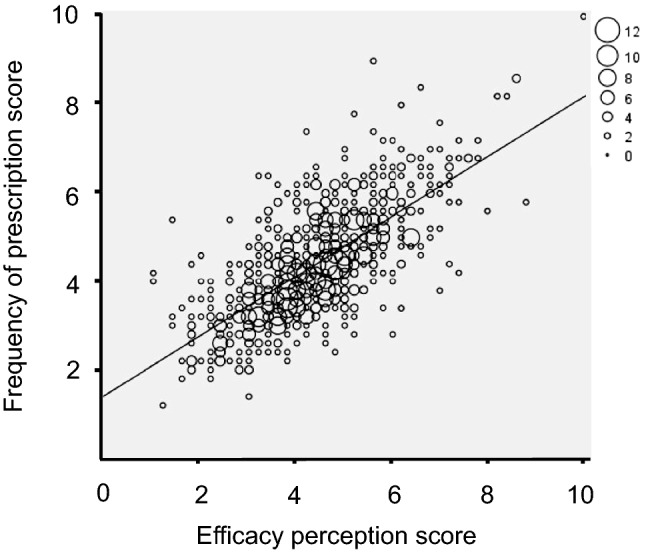
Correlation between frequency of prescription and perceived efficacy of treatments for refractory or unexplained chronic cough

Table 4 and Fig. 1 show the correlation between frequency of prescription and perceived effectiveness of the treatments used for refractory or unexplained chronic cough. The drugs with the lowest correlation were terpene derivatives (all 3 specialties), opioids (family physicians and pulmonologists), antihistamines (pulmonologists and allergists), antitussives and levodropropizone (allergists), and oral corticosteroids (pulmonologists). The correlation for all of the drugs grouped together suggested that around 45–47% of prescription was associated with perceived effectiveness, both for all 3 specialties together (ρ = 0.687, ρ^2^ = 0.472, figure) and separately for family physicians (ρ = 0.688, ρ^2^ = 0.473), pulmonologists (ρ = 0.665, ρ^2^ = 0.442), and allergists (ρ = 0.682, ρ^2^ = 0.465). Specific correlation studies by drug family and medical specialty suggested that prescription of antitussives and mucolytic agents by family physicians (supplementary Figs. 1 and 2) is higher than their perceived effectiveness, whereas for all specialists prescription of opioids (supplementary Fig. 3) and oral corticosteroids (supplementary Fig. 4) is lower with regard to their perceived effectiveness.

**Table 4:** Correlation between the scores assigned to the frequency of prescription and to the perceived effectiveness of the different treatments for refractory or unexplained chronic cough. Values are presented as the Spearman rho [ρ] with its square [ρ^2^]

	Family physicians (*n* = 620)	Pulmonologists (*n* = 92)	Allergists (*n* = 62)	All (*n* = 774)
	ρ (ρ^2^)	ρ (ρ^2^)	ρ (ρ^2^)	ρ (ρ^2^)
Antitussives^(1)^	0.640 (0.410)	0.674 (0.454)	0.581 (0.338)	0.644 (0.418)
Opioids^(2)^	0.545 (0.297)	0.446 (0.217)	0.673 (0.453)	0.563 (0.317)
Mucolytics^(3)^	0.756 (0.572)	0.683 (0.466)	0.723 (0.523)	0.748 (0.560)
Levodropropizine	0.650 (0.423)	0.699 (0.489)	0.488 (0.238)	0.653 (0.426)
Terpene derivatives	0.493 (0.243)	0.451 (0.203)	0.363 (0.132)	0.478 (0.228)
Antihistamines	0.723 (0.523)	0.625 (0.390)	0.554 (0.307)	0.697 (0.486)
Inhaled corticosteroids	0.684 (0.468)	0.715 (0.511)	0.638 (0.407)	0.670 (0.449)
Oral corticosteroids	0.652 (0.425)	0.561 (0.315)	0.633 (0.401)	0.632 (0.399)
Inhaled bronchodilators	0.728 (0.530)	0.689 (0.475)	0.701 (0.491)	0.708 (0.501)
Neuromodulators^(4)^	0.663 (0.440)	0.725 (0.526)	0.722 (0.521)	0.694 (0.482)

(1) dextromethorphan, cloperastine; (2) codeine, dimemorfan; (3) guaifenesin, acetylcysteine, ambroxol; (4) gabapentin, pregabalin

## Discussion

In this study, which was based on an anonymous survey completed by family physicians, pulmonologists, and allergists, we obtained data on the diagnostic tests performed to study patients with chronic cough and treatments prescribed to patients with refractory or unexplained chronic cough, as well as on perceived effectiveness of such treatments. Since the results are based on the direct responses of the physicians and not on a review of clinical records, they reflect the perceptions and opinions of these professionals. In addition, we analyzed the correlation between prescription and perception of efficacy scores assigned to different therapeutic families and found that around 45% of prescription was associated with perceived effectiveness; therefore, other factors may account for the more or less frequent prescription of drugs to treat unexplained or refractory chronic cough.

The diagnostic tool most widely used by family physicians and pulmonologists in patients with chronic cough was chest x-ray, as recommended in the ERS guidelines for the initial assessment of chronic cough [3]. These guidelines also recommend spirometry, although, according to our survey, its frequency of use is only notable among pulmonologists and allergists. All 3 specialties also frequently perform bronchodilator tests, which can suggest underlying asthma, although its value as a diagnostic tool for chronic cough remains unclear [8]. Pulmonologists and allergists also usually measure FeNO, a noninvasive technique that can reveal a possible underlying type 2 inflammatory mechanism mediated by eosinophils, as in eosinophilic bronchitis [9], and a potential response of cough to corticosteroids [10]. However, the clinical usefulness of FeNO for the diagnosis of chronic cough or for predicting the response to treatment has not yet been systematically investigated [11]. In addition to the limited access to certain diagnostic tests in primary care, the differences observed may be due to lack of scientific evidence or clear standardized protocols to diagnose and treat chronic cough, which might sometimes make coordination between different specialists more difficult.

Consistent with disease management guidelines, bronchodilators and inhaled corticosteroids are the drugs most frequently used by the 3 specialties to treat refractory or unexplained chronic cough [3]. Furthermore, the perceived effectiveness of these treatments was also high compared with the others analyzed. Although both bronchodilators and inhaled corticosteroids are the most widely used therapies by the respondents, evidence for using these agents in the treatment of chronic cough is scarce. For example, in the case of inhaled corticosteroids, some studies showed a certain benefit in terms of severity and frequency of cough compared with placebo [12, 13], whereas others revealed no differences, irrespective of whether patients had normal lung function or had chronic bronchitis or chronic obstructive pulmonary disease [14–17]. With a moderate level of evidence, the ERS guideline suggests using inhaled corticosteroids combined with a long-acting bronchodilator in patients with chronic cough and fixed airflow obstruction [3]. Also with a low level of evidence, the CHEST guideline stresses that inhaled corticosteroids should not be prescribed to adult patients with unexplained chronic cough and negative results in tests for bronchial hyperresponsiveness and eosinophilia (sputum eosinophils and FeNO) [18]. Given the scarce evidence available, the ERS guideline advises against the used of antacids and prokinetic agents [3].

Antihistamines are also used by the 3 specialists, albeit to a lesser extent. These agents could play a role when cough is associated with upper airway abnormalities, but empiric use in chronic cough is not supported by evidence. Family physicians, in particular, also reported prescribing antitussives, opioids, and mucolytics. A single-center study carried out over 4 weeks in 27 adult patients with refractory chronic cough reported moderate evidence—in the form of a significant 40% reduction in daily cough scores—for low-dose morphine (5–10 mg twice daily) [19]. Other neuromodulators that have been shown certain efficacy for the treatment of chronic cough are amitriptyline, gabapentin, tramadol, pregabalin, and baclofen [20–24]. However, scores recorded for prescription and perceived effectiveness of neuromodulators were very low, maybe due to the lack of indication for chronic cough and the frequency of adverse effects when used at doses effective for inhibiting cough. In fact, the CHEST guidelines suggest informing patients with chronic cough about the possible adverse effects and risk–benefit profile of these agents [18].

The correlation analysis for prescription and perceived effectiveness showed that around 45% of prescription was related to perceived effectiveness, suggesting that other factors may account for greater or lesser prescription of drugs for chronic cough. In fact, some therapeutic agents might be prescribed less frequently owing to adverse events, and others with a limited efficacy profile but better safety profile may be prescribed more often. In our study, opioids and oral corticosteroids seem to be prescribed less frequently by all specialists despite their perceived effectiveness (probably as a consequence of their safety profile), whereas antitussives and mucolytics, which are easier to manage, seem to be prescribed more often in primary care than their perceived effectiveness would suggest.

Our findings highlight the need for clear diagnostic and treatment protocols and for effective treatment of refractory or unexplained/idiopathic chronic cough. Improved knowledge of the neurogenic pathways of cough and of the neurophysiological abnormalities underlying refractory or unexplained chronic cough has made it possible to develop drugs that block these pathways, such as sodium channel blockers [25], transient receptor potential vanilloid 1 antagonists (TRPV1) [26, 27], and P2X3 receptor antagonists [28, 29].

Our study is subject to the limitations inherent to surveys. First, since we have no information on the profile of the physicians who did not respond, the perceptions of the respondents may not reflect majority opinion. Given its voluntary nature, the survey may have been completed by physicians who were particularly interested in the condition studied. Survey participation rate was low (1.9% of family physicians and 4.1% of pulmonologists and allergists), and the number of family physicians who completed the survey was significantly higher than pulmonologists and allergists. Although participants declared to see a substantial number of patients with chronic cough at their clinics, their opinions could not be representative of other colleagues.

Second, participants responded to the survey based on their perceptions and not on the review of clinical records. Thus, there was no data source verification. Besides, there are several facts inherent to clinical practice in Spain that can have conditioned physicians’ responses and must be considered when interpreting the results. Reasons for the different responses were not requested (e.g., why a specific diagnostic test is not performed or why a particular treatment is prescribed or not). Family physicians do not have access, or have limited access to methacholine test, FeNO test, capsaicin test, specific IgE, and esophageal pH monitoring. They request these tests to specific specialists (pulmonologists, allergists, or gastroenterologists). In general, pulmonologists and allergists have access to results of tests performed by family physicians. If they rely on tests already performed in primary care, they will not need to indicate those tests again to chronic cough patients. These facts can have conditioned the responses by family physicians, pulmonologists, and allergists. In addition, the survey reflects mostly the opinion of physicians working in the public national health system; only about 5% of respondents declared to work only in the private sector, where clinical practice could differ. Most respondents used SEPAR guidelines for the management of chronic cough. This information should be noted when comparing the results with other surveys in Europe and worldwide.

Finally, the survey did not include separate questions for refractory and for unexplained chronic cough. Although this does not affect outcomes on the different diagnostic tests (the question was for the study of chronic cough in general), we cannot exclude differences in therapies used and perceived effectiveness (Tables 2 and 3) between refractory and unexplained chronic cough.

With all these limitations, however, the results obtained help us to understand the diagnosis and treatment of chronic cough by these three specialists in Spain and can serve as a starting point from which to standardize and improve patient care.

## Conclusion

Chronic cough is one of the most frequent reasons for visits to healthcare providers. However, little is known about the perceptions of the specialists who treat this complaint with respect to diagnostic techniques for the study of chronic cough and therapies used for the treatment of refractory or unexplained chronic cough. The present study reports differences in diagnosis and treatment between the 3 specialties. Perception of the effectiveness of available drugs is generally low, except for inhaled drugs and oral corticosteroids, and the association between perceived effectiveness and prescription shows that the latter is not only associated with perceived effectiveness, but also with other factors (probably ease of use and safety profile). It is important to have diagnostic and treatment protocols agreed upon by the different specialties in order to standardize the approach to patients with chronic cough.

## Supplementary Information

Below is the link to the electronic supplementary material.

Supplementary file1 (PPTX 257 KB)

Supplementary file2 (DOCX 42 KB)

## Data Availability

Not applicable.

## References

[1] Chamberlain SA, Garrod R, Douiri A (2015). The impact of chronic cough: a cross-sectional European survey. Lung.

[2] Song WJ, Chang YS, Faruqi S (2015). The global epidemiology of chronic cough in adults: a systematic review and meta-analysis. Eur Respir J.

[3] Morice AH, Millqvist E, Bieksiene K (2020). ERS guidelines on the diagnosis and treatment of chronic cough in adults and children. Eur Respir J.

[4] Morice AH, Millqvist E, Belvisi MG (2014). Expert opinion on the cough hypersensitivity syndrome in respiratory medicine. Eur Respir J.

[5] Mazzone SB, Chung KF, McGarvey L (2018). The heterogeneity of chronic cough: a case for endotypes of cough hypersensitivity. Lancet Respir Med.

[6] Chung KF, McGarvey L, Mazzone SB (2013). Chronic cough as a neuropathic disorder. Lancet Respir Med.

[7] Chung KF, McGarvey L, Mazzone S (2016). Chronic cough and cough hypersensitivity syndrome. Lancet Respir Med.

[8] McGarvey LP, Heaney LG, Lawson JT (1998). Evaluation and outcome of patients with chronic non-productive cough using a comprehensive diagnostic protocol. Thorax.

[9] Brightling CE, Ward R, Goh KL (1999). Eosinophilic bronchitis is an important cause of chronic cough. Am J Respir Crit Care Med.

[10] Lamon T, Didier A, Brouquieres D (2019). Exhaled nitric oxide in chronic cough: A good tool in a multi-step approach. Respir Med Res.

[11] Sadeghi MH, Wright CE, Hart S (2018). Phenotyping patients with chronic cough: evaluating the ability to predict the response to anti-inflammatory therapy. Ann Allergy Asthma Immunol.

[12] Chaudhuri R, McMahon AD, Thomson LJ (2004). Effect of inhaled corticosteroids on symptom severity and sputum mediator levels in chronic persistent cough. J Allergy Clin Immunol.

[13] Ribeiro M, Pereira CA, Nery LE (2007). High-dose inhaled beclomethasone treatment in patients with chronic cough: a randomized placebo-controlled study. Ann Allergy Asthma Immunol.

[14] Boulet LP, Milot J, Boutet M (1994). Airway inflammation in nonasthmatic subjects with chronic cough. Am J Respir Crit Care Med.

[15] Pizzichini MM, Pizzichini E, Parameswaran K (1999). Nonasthmatic chronic cough: no effect of treatment with an inhaled corticosteroid in patients without sputum eosinophilia. Can Respir J.

[16] Wesseling GJ, Quaedvlieg M, Wouters EF (1991). Inhaled budesonide in chronic bronchitis. effects on respiratory impedance. Eur Respir J.

[17] Calverley P, Pauwels R, Vestbo J (2003). Combined salmeterol and fluticasone in the treatment of chronic obstructive pulmonary disease: a randomised controlled trial. Lancet.

[18] Gibson P, Wang G, McGarvey L (2016). Treatment of unexplained chronic cough: chest guideline and expert panel report. Chest.

[19] Morice AH, Menon MS, Mulrennan SA (2007). Opiate therapy in chronic cough. Am J Respir Crit Care Med.

[20] Jeyakumar A, Brickman TM, Haben M (2006). Effectiveness of amitriptyline versus cough suppressants in the treatment of chronic cough resulting from postviral vagal neuropathy. Laryngoscope.

[21] Ryan NM, Birring SS, Gibson PG (2012). Gabapentin for refractory chronic cough: a randomised, double-blind, placebo-controlled trial. Lancet.

[22] Dion GR, Teng SE, Achlatis E, Fang Y (2017). Treatment of neurogenic cough with tramadol: a pilot study. Otolaryngol Head Neck Surg.

[23] Cohen SM, Misono S (2013). Use of specific neuromodulators in the treatment of chronic, idiopathic cough: a systematic review. Otolaryngol Head Neck Surg.

[24] Wei W, Liu R, ZhangTong Y et al (2016) The efficacy of specific neuromodulators on human refractory chronic cough: a systematic review and meta-analysis. J Thorac Dis 8: 2942–2951 Doi: 10.21037/jtd.2016.10.51 10.21037/jtd.2016.10.51 PMC5107493 27867572

[25] Smith JA, McGarvey LPA, Badri H (2017). Effects of a novel sodium channel blocker, GSK2339345, in patients with refractory chronic cough. Int J Clin Pharmacol Ther.

[26] Belvisi MG, Birrell MA, Wortley MA (2017). XEN-D0501, a novel transient receptor potential vanilloid 1 antagonist, does not reduce cough in patients with refractory cough. Am J Respir Crit Care Med.

[27] Khalid S, Murdoch R, Newlands A (2014). Transient receptor potential vanilloid 1 (TRPV1) antagonism in patients with refractory chronic cough: a double-blind randomized controlled trial. J Allergy Clin Immunol.

[28] Muccino D, Green S (2019). Update on the clinical development of gefapixant, a P2X3 receptor antagonist for the treatment of refractory chronic cough. Pulm Pharmacol Ther.

[29] Smith JA, Kitt MM, Morice AH (2020). Gefapixant, a P2X3 receptor antagonist, for the treatment of refractory or unexplained chronic cough: a randomised, double-blind, controlled, parallel-group, phase 2b trial. Lancet Respir Med.

