# Supplementary material for: A Survey of Physicians' Perception of the Use and Effectiveness of Diagnostic and Therapeutic Procedures in Chronic Cough Patients
Source: Lung. 2021 Sep 17;199(5):507–15. doi: 10.1007/s00408-021-00475-1 (PMC8510925; doi:10.1007/s00408-021-00475-1)
Supplement: Supplementary file 1 — Supplementary file1 (PPTX 257 KB) [file 408_2021_475_MOESM1_ESM.pptx]

## Slide 1
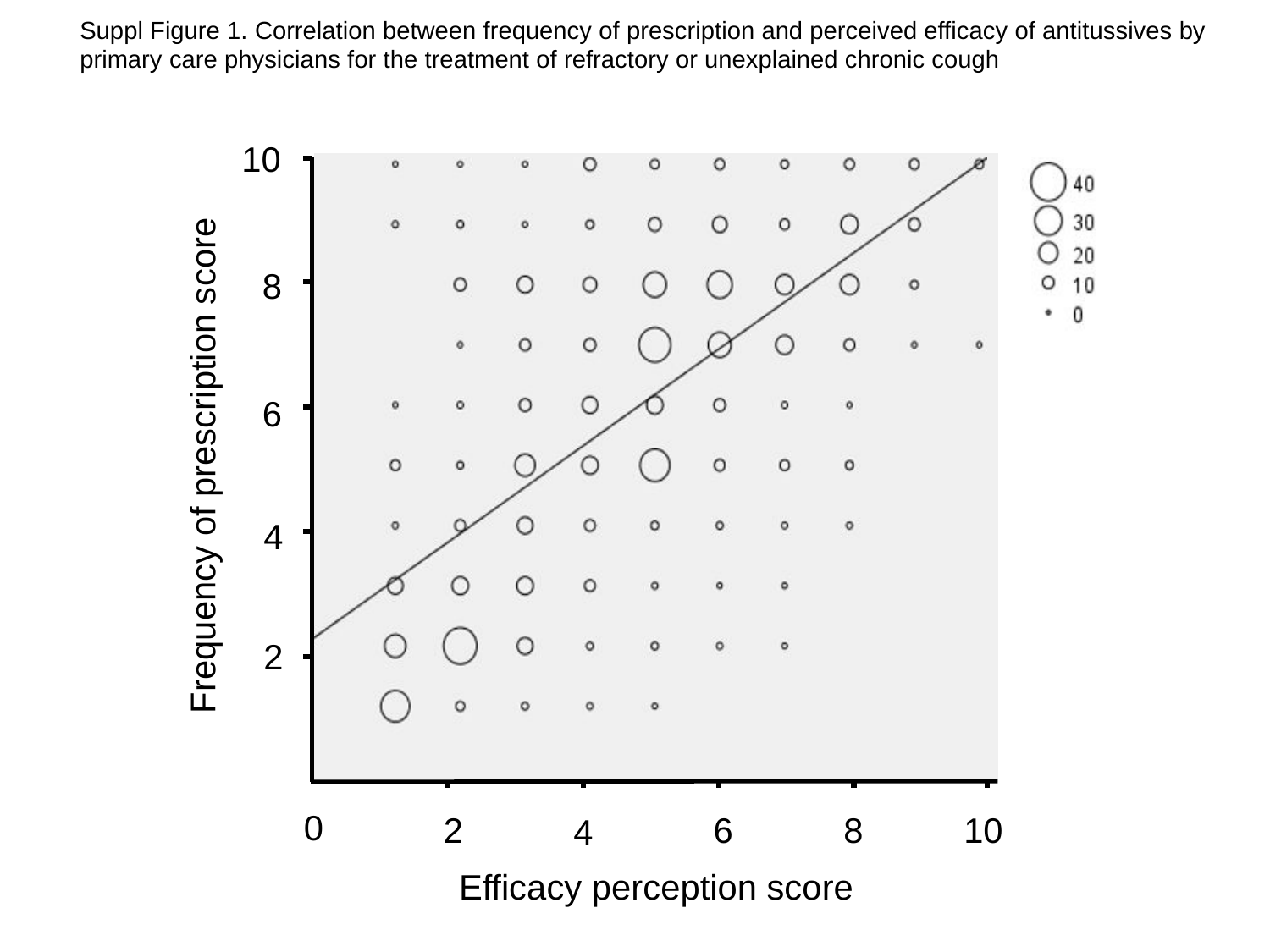

Suppl Figure 1. Correlation between frequency of prescription and perceived efficacy of antitussives by primary care physicians for the treatment of refractory or unexplained chronic cough
10
8
6
4
2
0
2
6
8
10
4
Frequency of prescription score
Efficacy perception score

## Slide 2
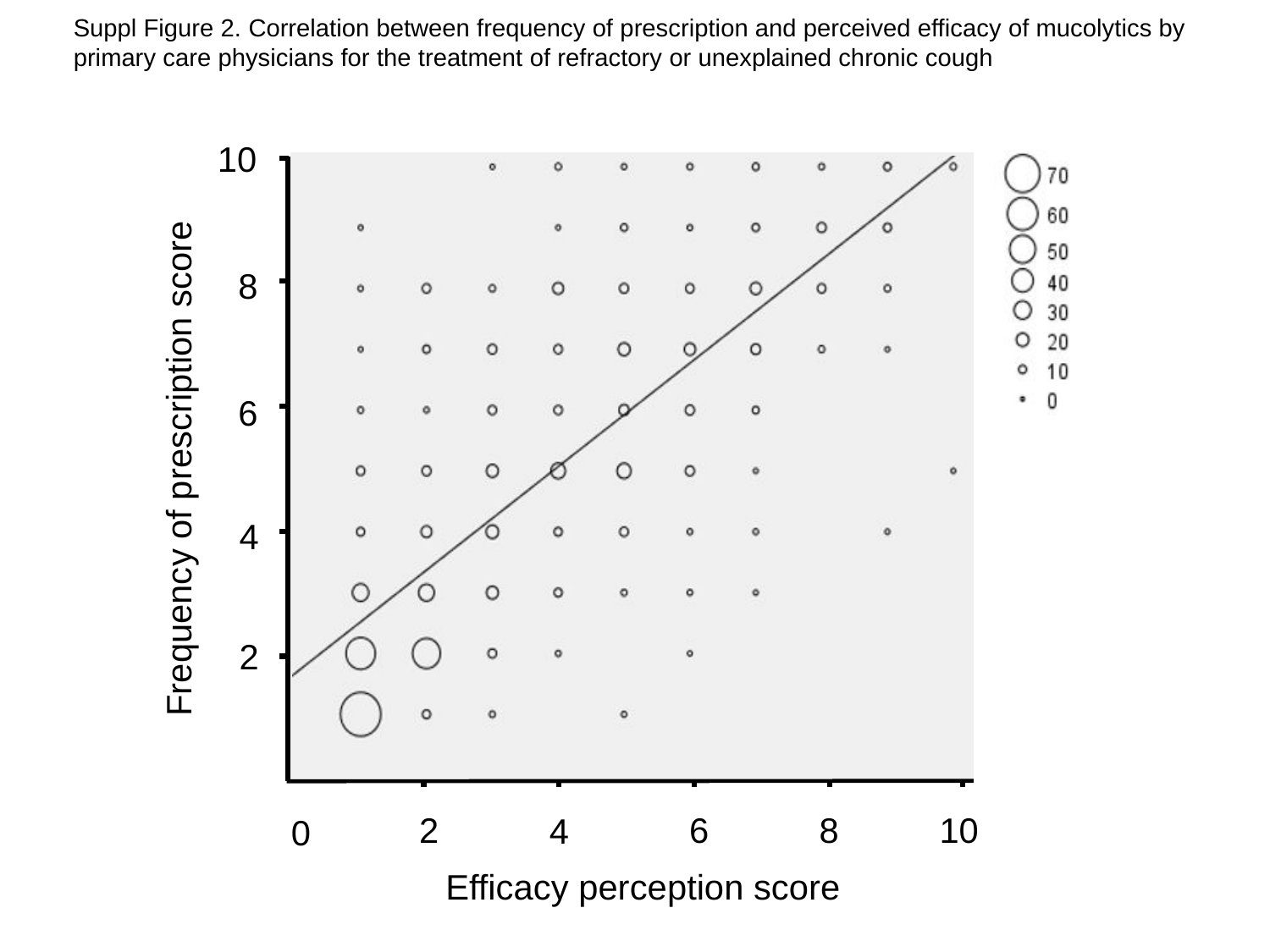

Suppl Figure 2. Correlation between frequency of prescription and perceived efficacy of mucolytics by primary care physicians for the treatment of refractory or unexplained chronic cough
10
8
6
4
2
2
6
8
10
4
0
Frequency of prescription score
Efficacy perception score

## Slide 3
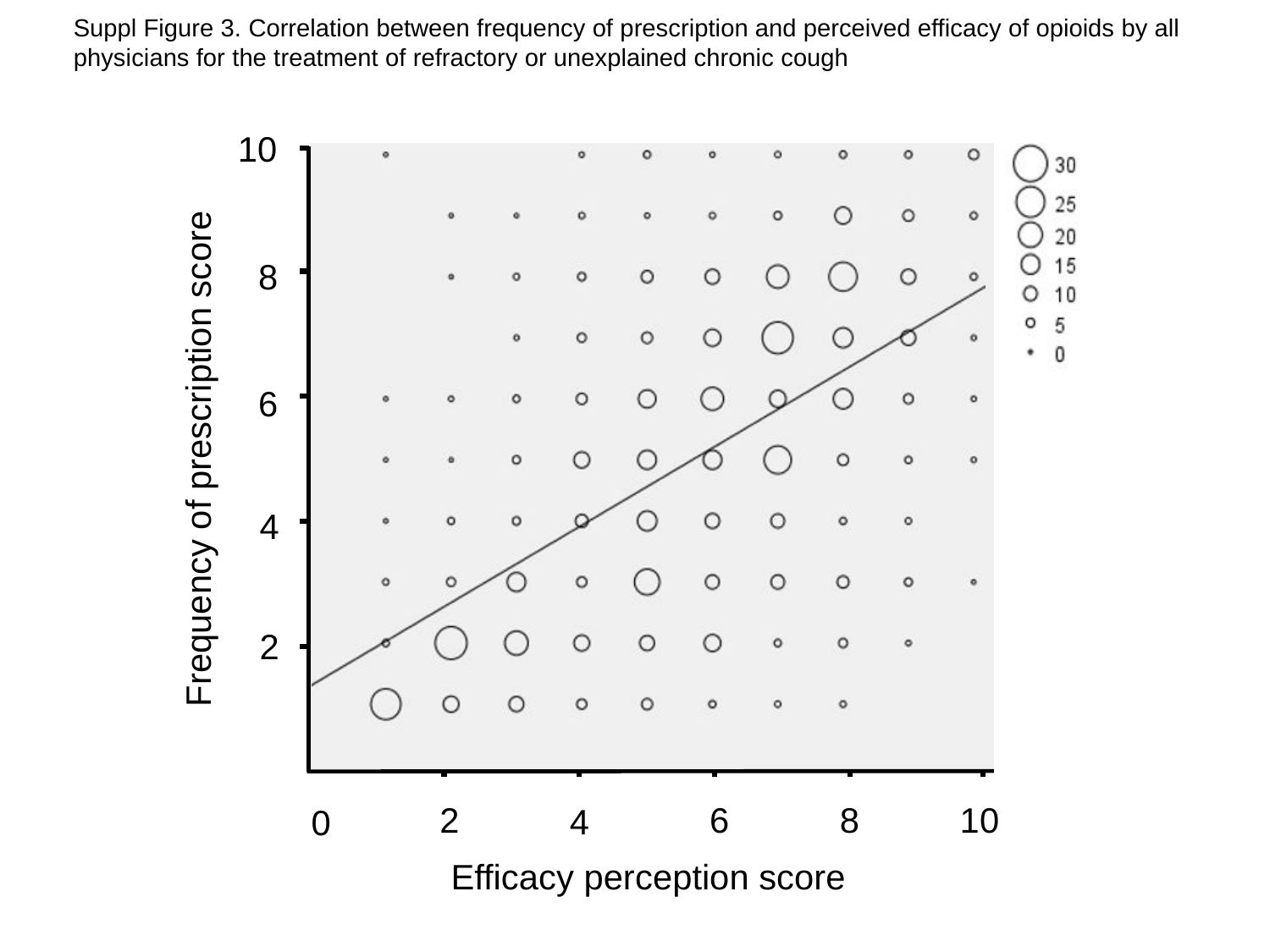

Suppl Figure 3. Correlation between frequency of prescription and perceived efficacy of opioids by all physicians for the treatment of refractory or unexplained chronic cough
10
8
6
4
2
2
6
8
10
4
0
Frequency of prescription score
Efficacy perception score

## Slide 4
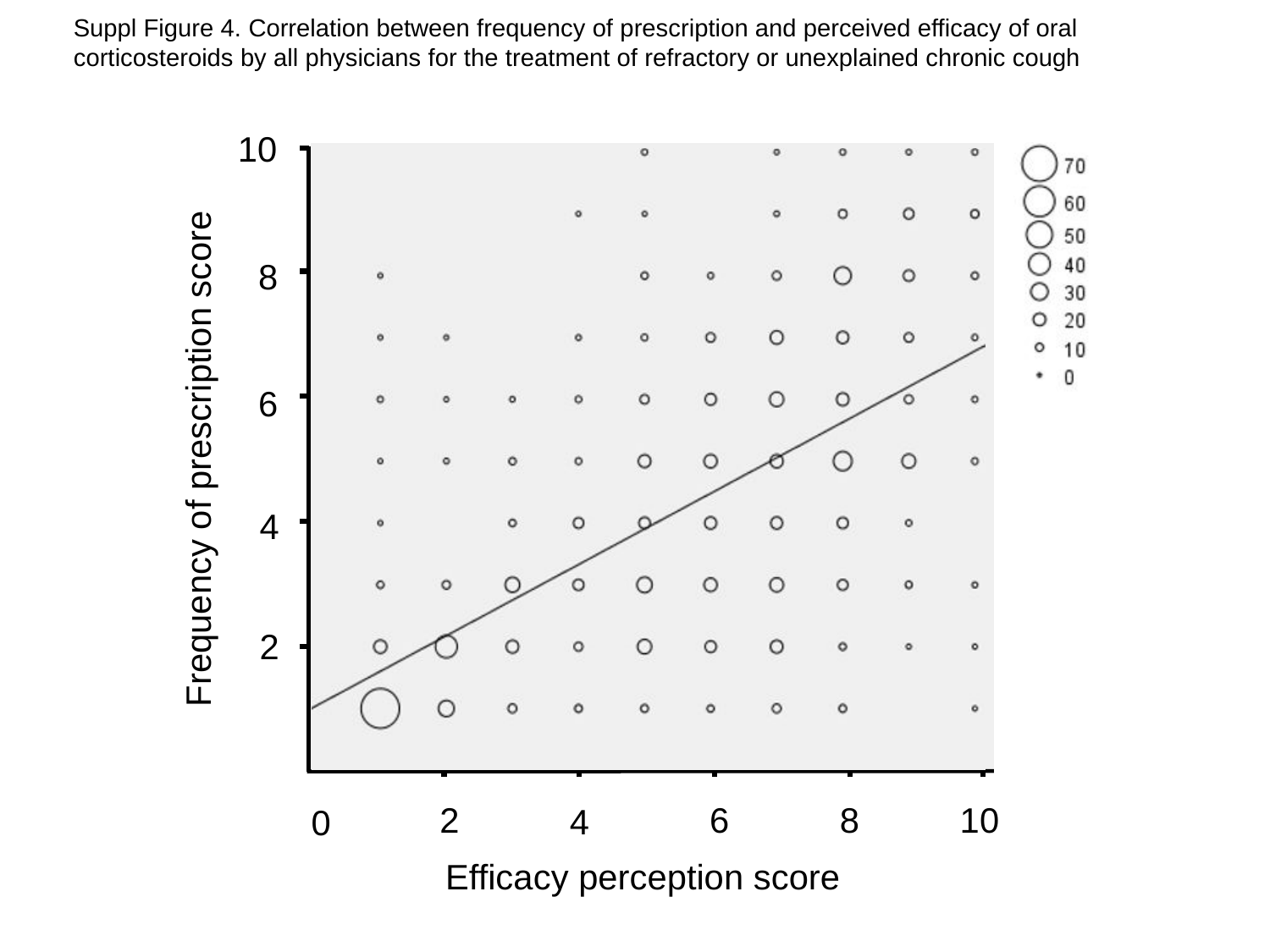

Suppl Figure 4. Correlation between frequency of prescription and perceived efficacy of oral corticosteroids by all physicians for the treatment of refractory or unexplained chronic cough
10
8
6
4
2
2
6
8
10
4
0
Frequency of prescription score
Efficacy perception score
