# Supplementary material for: A Survey of Physicians' Perception of the Use and Effectiveness of Diagnostic and Therapeutic Procedures in Chronic Cough Patients
Source: Lung. 2021 Sep 17;199(5):507–15. doi: 10.1007/s00408-021-00475-1 (PMC8510925; doi:10.1007/s00408-021-00475-1)
Supplement: Supplementary file 2 — Supplementary file2 (DOCX 42 KB) [file 408_2021_475_MOESM2_ESM.docx]

**SURVEY**

1. Number of patients seen weekly at your clinic (average of patients -whatever the reason- seen in a week -new patients and revisions-)

- Less than 50 patients / week
- Between 50-100 patients / week
- Between 100-200 patients / week
- Between 200-300 patients / week
- More than 300 patients / week

1. Among all patients you see at your clinic, chronic cough represents:

- <5% of patients
- Between 5-10% of patients
- Between 10-20% of patients
- Between 20-50% of patients
- •> 50% of patients

1. Thinking about the last week, please indicate the number of patients with Chronic Cough of any etiology that you have seen in your consultation.

(number)

1. When do you state a diagnosis of chronic cough in a patient with a cough?

- When cough lasts more than 4 weeks
- When cough lasts more than 8 weeks
- When cough lasts more than 12 weeks
- I don't usually have a specific criterion
- I don't see this type of patients

1. Regarding the diagnostic work-up of patients with chronic cough, indicate the frequency with which you perform the following examinations routinely. Use a Scale from 1 to 10; (1 = I never perform the test; 10 = I always use the test)

|  | 1 | 2 | 3 | 4 | 5 | 6 | 7 | 8 | 9 | 10 |
| --- | --- | --- | --- | --- | --- | --- | --- | --- | --- | --- |
| Chest x-ray |  |  |  |  |  |  |  |  |  |  |
| Simple spirometry |  |  |  |  |  |  |  |  |  |  |
| Bronchodilator test |  |  |  |  |  |  |  |  |  |  |
| Methacholine test |  |  |  |  |  |  |  |  |  |  |
| FeNO test |  |  |  |  |  |  |  |  |  |  |
| Capsaicin test |  |  |  |  |  |  |  |  |  |  |
| Complete blood count |  |  |  |  |  |  |  |  |  |  |
| Total IgE |  |  |  |  |  |  |  |  |  |  |
| Specific IgE |  |  |  |  |  |  |  |  |  |  |
| *Chlamydia/Mycoplasma* serology |  |  |  |  |  |  |  |  |  |  |
| Esophageal pH monitoring |  |  |  |  |  |  |  |  |  |  |

1. Think about the patients with chronic cough that you see at your clinic, and indicate, in your experience, how often are the underlying diseases as a cause of chronic cough.

|  | Never | Rarely | Sometimes | Often |
| --- | --- | --- | --- | --- |
| No other underlying disease |  |  |  |  |
| Psychological disorders |  |  |  |  |
| Asthma |  |  |  |  |
| Rhinosinusitis |  |  |  |  |
| Gastroesophageal reflux |  |  |  |  |
| Chronic obstructive pulmonary disease |  |  |  |  |
| Smoking |  |  |  |  |
| Eosinophilic bronchitis |  |  |  |  |
| Treatment with angiotensin converting enzyme inhibitors (ACE inhibitors) |  |  |  |  |

1. Please indicate how often you use the following terms for the diagnosis of patients with chronic cough

|  | Never | Rarely | Sometimes | Often |
| --- | --- | --- | --- | --- |
| Unexplained/idiopathic chronic cough |  |  |  |  |
| Refractory chronic cough |  |  |  |  |
| Cough hypersensitivity syndrome |  |  |  |  |

1. Based on your experience in the management of patients with chronic cough (refractory or unexplained), indicate your degree of agreement with the following statements

|  | Strongly disagree | Disagree | Agree | Strongly agree |
| --- | --- | --- | --- | --- |
| Refractory chronic cough is a symptom related to a respiratory underlying disease |  |  |  |  |
| Refractory chronic cough is a symptom related to a non-respiratory underlying disease |  |  |  |  |
| Refractory/unexplained chronic cough is a disease in itself |  |  |  |  |

1. After having performed the available diagnostic tests, if the patient has refractory or unexplained chronic cough, what is your general behavior?

NOTE = THIS QUESTION VARIES DEPENDING ON THE SPECIALIST ADRESSED (for example, allergists do not see the option “I refer the patient to the allergist”)

|  | Never | Rarely | Sometimes | Often |
| --- | --- | --- | --- | --- |
| I start treatment and I assume the follow-up of the patient |  |  |  |  |
| I start treatment and refer the patient to the pulmonologist for follow-up |  |  |  |  |
| I start treatment and refer the patient to the allergist for follow-up |  |  |  |  |
| I start treatment and refer the patient to the family physician for follow-up |  |  |  |  |
| I refer to other specialist without initiating any treatment |  |  |  |  |

1. Regarding the treatment of patients with refractory or unexplained chronic cough: indicate the frequency with which you prescribe each of these drugs. Use a Scale from 1 to 10 (1 = I never prescribe the drug; 10 = I always prescribe the drug)

|  | 1 | 2 | 3 | 4 | 5 | 6 | 7 | 8 | 9 | 10 |
| --- | --- | --- | --- | --- | --- | --- | --- | --- | --- | --- |
| Antitussives^(1)^ |  |  |  |  |  |  |  |  |  |  |
| Opioids^(2)^ |  |  |  |  |  |  |  |  |  |  |
| Mucolytics^(3)^ |  |  |  |  |  |  |  |  |  |  |
| Levodropropizine |  |  |  |  |  |  |  |  |  |  |
| Terpene derivatives |  |  |  |  |  |  |  |  |  |  |
| Antihistamines |  |  |  |  |  |  |  |  |  |  |
| Inhaled corticosteroids |  |  |  |  |  |  |  |  |  |  |
| Oral corticosteroids |  |  |  |  |  |  |  |  |  |  |
| Inhaled bronchodilators |  |  |  |  |  |  |  |  |  |  |
| Neuromodulators^(4)^ |  |  |  |  |  |  |  |  |  |  |

(1) dextromethorphan, cloperastine; (2) codeine, dimemorfan; (3) guaifenesin, acetylcysteine, ambroxol; (4) gabapentin, pregabalin.

1. Based on your experience treating patients with chronic cough (refractory or unexplained), please indicate your perception of the efficacy of each of the following drugs. Use a Scale from 1 to 10 (1 = Not effective at all; 10 = Most effective)

|  | 1 | 2 | 3 | 4 | 5 | 6 | 7 | 8 | 9 | 10 |
| --- | --- | --- | --- | --- | --- | --- | --- | --- | --- | --- |
| Antitussives^(1)^ |  |  |  |  |  |  |  |  |  |  |
| Opioids^(2)^ |  |  |  |  |  |  |  |  |  |  |
| Mucolytics^(3)^ |  |  |  |  |  |  |  |  |  |  |
| Levodropropizine |  |  |  |  |  |  |  |  |  |  |
| Terpene derivatives |  |  |  |  |  |  |  |  |  |  |
| Antihistamines |  |  |  |  |  |  |  |  |  |  |
| Inhaled corticosteroids |  |  |  |  |  |  |  |  |  |  |
| Oral corticosteroids |  |  |  |  |  |  |  |  |  |  |
| Inhaled bronchodilators |  |  |  |  |  |  |  |  |  |  |
| Neuromodulators^(4)^ |  |  |  |  |  |  |  |  |  |  |

(1) dextromethorphan, cloperastine; (2) codeine, dimemorfan; (3) guaifenesin, acetylcysteine, ambroxol; (4) gabapentin, pregabalin.

1. Please indicate your degree of agreement with each of the statements related to the evolution of refractory/unexplained chronic cough in the patients you usually see at your clinic. (Scale 1-10- 1: Totally disagree / 10: Totally agree)

|  | 1 | 2 | 3 | 4 | 5 | 6 | 7 | 8 | 9 | 10 |
| --- | --- | --- | --- | --- | --- | --- | --- | --- | --- | --- |
| It usually disappears by itself after a while |  |  |  |  |  |  |  |  |  |  |
| It does not usually disappear by itself; it persists over time |  |  |  |  |  |  |  |  |  |  |
| If there is an underlying disease, it disappears when treating it |  |  |  |  |  |  |  |  |  |  |
| Even if there is an underlying disease, it does not disappear despite treating it |  |  |  |  |  |  |  |  |  |  |
| I'm not sure how chronic cough evolves |  |  |  |  |  |  |  |  |  |  |

1. Please indicate the frequency with which you refer patients with refractory/unexplained chronic cough to be seen by another specialist

NOTE = THIS QUESTION VARIES DEPENDING ON THE SPECIALIST ADRESSED (for example, allergists do not see the option “I refer the patient to the allergist”)

|  | Never | Rarely | Sometimes | Often |
| --- | --- | --- | --- | --- |
| I refer the patient to the pulmonologist |  |  |  |  |
| I refer the patient to the allergist |  |  |  |  |
| I refer the patient to the family physician |  |  |  |  |
| I refer the patient to the ETN specialist |  |  |  |  |
| I refer the patient to the gastroenterologist |  |  |  |  |
| I refer the patient to the psychiatrist |  |  |  |  |

1. Thinking about the follow-up of patients with refractory/unexplained chronic cough, indicate the frequency with which you think the different specialties undertake the follow-up of these patients after all the diagnostic tests have been done

|  | Never | Rarely | Sometimes | Often |
| --- | --- | --- | --- | --- |
| Patients are usually followed by the pulmonologist |  |  |  |  |
| Patients are usually followed by the allergist |  |  |  |  |
| Patients are usually followed by the family physician |  |  |  |  |
| Patients are usually followed by the ETN specialist |  |  |  |  |
| Patients are usually lost for follow-up |  |  |  |  |

1. How often do you use the following guidelines for the management of chronic cough to diagnose and treat your patients?

|  | Never | Rarely | Sometimes | Often |
| --- | --- | --- | --- | --- |
| American College of Chest Physicians guidelines |  |  |  |  |
| European Respiratory Society guidelines |  |  |  |  |
| British Thoracic Society guidelines |  |  |  |  |
| Normativa SEPAR for the management of chronic cough (Spanish Society of Respiratory Medicine) |  |  |  |  |

1. Please indicate you have a protocol for the diagnosis and treatment of chronic cough available at your work center

|  | Yes | No |
| --- | --- | --- |
| Protocol for the diagnosis of patients with chronic cough |  |  |
| Protocol for the treatment of patients with chronic cough |  |  |

1. To what extent do you consider necessary the existence of Protocols for the diagnosis and treatment of chronic cough in your workplace?

|  | Not at all | Slightly necessary | Quite necessary | Very much necessary |
| --- | --- | --- | --- | --- |
| Protocol for the diagnosis of patients with chronic cough |  |  |  |  |
| Protocol for the treatment of patients with chronic cough |  |  |  |  |

1. Please indicate your degree of agreement with each of the following statements regarding refractory/unexplained chronic cough

|  | Strongly disagree | Disagree | Agree | Strongly agree |
| --- | --- | --- | --- | --- |
| Is generally associated with urinary incontinence |  |  |  |  |
| Is more frequent in women than in men |  |  |  |  |
| Is more frequent in older people |  |  |  |  |
| Is more frequent in people with a low socio-cultural level |  |  |  |  |
| Is generally associated with chronic pain |  |  |  |  |
| Is generally associated with psychological disorders |  |  |  |  |

1. Please indicate from 1 to 10 the impact that, in your opinion, the following diseases have on the quality of life of patients. (1 = it has no impact; 10 = it has a very high impact)

|  | 1 | 2 | 3 | 4 | 5 | 6 | 7 | 8 | 9 | 10 |
| --- | --- | --- | --- | --- | --- | --- | --- | --- | --- | --- |
| Rhinitis |  |  |  |  |  |  |  |  |  |  |
| Chronic cough |  |  |  |  |  |  |  |  |  |  |
| Asthma |  |  |  |  |  |  |  |  |  |  |
| COPD |  |  |  |  |  |  |  |  |  |  |
| Diabetes |  |  |  |  |  |  |  |  |  |  |
| Migraine |  |  |  |  |  |  |  |  |  |  |

1. Regarding patients with refractory/unexplained chronic cough, please indicate the impact you think chronic cough has on the following aspects. (1 = chronic cough has no impact; 10 = chronic cough has a very high impact)

|  | 1 | 2 | 3 | 4 | 5 | 6 | 7 | 8 | 9 | 10 |
| --- | --- | --- | --- | --- | --- | --- | --- | --- | --- | --- |
| Psychological aspects: anxiety / depression |  |  |  |  |  |  |  |  |  |  |
| Leisure and free time activities |  |  |  |  |  |  |  |  |  |  |
| Sleep |  |  |  |  |  |  |  |  |  |  |
| Emotional sphere |  |  |  |  |  |  |  |  |  |  |
| Physical activity / exercise |  |  |  |  |  |  |  |  |  |  |
| Family life |  |  |  |  |  |  |  |  |  |  |
| Social life |  |  |  |  |  |  |  |  |  |  |
| Private/sexual life |  |  |  |  |  |  |  |  |  |  |
| Work activity |  |  |  |  |  |  |  |  |  |  |

1. Please indicate your age (years old)

- <35
- 36-45
- 46-55
- 56-65
- > 65

1. Please indicate your sex

- Man
- Woman

1. Years of Experience (not counting residence or fellowships)

- <5
- 5-15
- > 15

1. In what type of clinic do you carry out your professional activity (you can choose both options)?

- Public
- Private
